# Health status, difficulties, and desired health information and services for veterans with traumatic brain injuries and their caregivers: A qualitative investigation

**DOI:** 10.1371/journal.pone.0203804

**Published:** 2018-09-12

**Authors:** Christine Koehmstedt, Susan E. Lydick, Drasti Patel, Xinsheng Cai, Steven Garfinkel, Ali A. Weinstein

**Affiliations:** 1 Center for the Study of Chronic Illness and Disability, College of Health and Human Services, George Mason University, Fairfax, Virginia, United States of America; 2 American Institutes for Research, Washington, DC, United States of America; Public Library of Science, UNITED KINGDOM

## Abstract

Traumatic brain injury (TBI) is considered the signature injury among military service member and Veterans who served in Operation Iraqi Freedom and Operation Enduring Freedom with over 360,000 individuals sustaining a first-time TBI in the military. These service members and Veterans, and their caregiver(s), must navigate multiple health systems and find experts across many fields of expertise to recover and optimize functionality. Twenty-two individuals, 10 caregivers of Veterans with TBI, 12 Veterans with TBI, participated in semi-structured interviews. Responses were coded using NVivo. Participants from both groups reported difficulties finding community supportive services (support groups) in local communities. Most participants identified the need for an advocate or point-person to help guide them to needed services and provide ongoing support in the post-acute health care recovery phase. Caregivers and Veterans desired a more personalized recovery plan from their medical professionals. When describing their ideal health information and services model most identified interactivity and twenty-four-hour availability as essential components. To provide Veterans and caregivers with optimal support and resources to navigate a complicated health services system, advocates and personalized care plans are needed. Future research should examine the feasibility and cost-effectiveness of these services.

## Introduction

Traumatic Brain Injury (TBI) is being labeled the signature injury of Operation Iraqi Freedom (OIF) and Operation Enduring Freedom (OEF). Since 2000, 361,092 service members have sustained a first-time TBI in the military [1,2]. This specific injury often impacts multiple body systems yet remains an “invisible” injury. Common symptoms of TBI are any combination of headaches, confusion, agitation, slurred speech, fatigue, sleep disturbance, vestibular disturbances, sensory problems, memory concentration difficulties, irritability, aggression, vomiting or nausea, problems with judgment and executive control, and convulsions or seizures [3,4]

The recovery periods vary greatly for these military service members and Veterans. Military service members who screen positive for mild TBI (mTBI) are more likely to experience symptoms (i.e., sleep problems, forgetfulness, headaches) than those that did not suffer a mTBI, after three months postdeployment [5] and after nine months postdeployment [6]. And, these symptoms can last longer with an estimated 10 to 15 percent of service members report experiencing ongoing difficulty with reintegration and experiencing psychological challenges for long periods of time [7]. For those who sustained a moderate to severe TBI, recovery is much more varied and unpredictable. Some may return to baseline functionality quickly, however, many experience permanent changes in health and functionality [8].

Given the high variability in the recovery process, it is difficult to estimate the number of military service members and Veterans who sustained a TBI in recent wars, who are now faced with learning to adapt to lifelong health challenges. This variability regarding impact of injury on health and functioning is likely multi-determined by factors including cause of TBI (blast, closed head blunt force, or penetrating wound), site(s) of injury within the brain, and severity of the TBI (mild to severe) [9–11].

Prior to OIF/OEF, TBI was viewed as a stable condition after the acute state. Health professionals now recognize TBI as a chronic condition with health challenges that are sustained over long periods of time (psychological, somatic, and cognitive) [12–14]. Furthermore, adding increased complexity to Veteran health status, persons who sustained a TBI in combat may have sustained additional physical injuries (bone fractures, amputations, burns, spinal cord injuries) as well as psychological trauma resulting in PTSD [15]. Thus, multiple body systems are likely impacted directly by the TBI in addition to the physical and psychological traumas experienced. As a result, these Veterans are faced with navigating symptoms that may change over time, both with age and with major life transitions such as transition from military to Veteran status [16–18].

Many of these Veterans and their caregiver(s) or other advocate(s) will need to seek long-term care from a variety of health care providers and experts, across multiple health care systems (Department of Defense/DOD, Veterans Administration/VA, Civilian). This will span multiple transitions throughout their life to achieve optimal quality of life with a TBI. To cope with these transitions, it is important to understand the supportive services and up to date health information that the Veterans and caregivers will need. In addition, Veterans and caregivers will need to know strategies to help them on a daily basis cope and adapt to their own particular set of symptoms, health challenges, and functional limitations [19,20].

Prescribing appropriate strategies to help Veterans and caregivers adapt and cope once leaving an acute health care setting is difficult. Symptoms associated with TBI may manifest and be different at home than in a protective acute care or subsequent rehabilitation setting [21]. Additionally, these symptoms may change over time. Therefore, although Veterans and caregivers may receive plans upon leaving the hospital, continuing education and ongoing support for both the Veteran and caregiver is critical to meet the challenges of living with TBI.

To date, the DOD and the VA have responded to this service delivery challenge primarily through offering numerous educational websites. These websites encourage Veterans and caregivers to access information about coping with and adapting to life with TBI [22]. Several other websites include the Defense and Veterans Brain Injury Center, National Intrepid Center of Excellence, and the Journey Home with TBI [23–25]. These websites and others offer downloadable factsheets, booklets, educational videos, and podcasts about living with TBI and caring for Veterans with TBI.

In recognition of Veteran and caregiver need for individualized information and consultation to support progress in their TBI recovery, in 2007 DOD and VA developed the Recovery Support Specialist (RSS) program (previously known as the Care Coordination Program) [23]. The role of the RSS is to follow the Veteran throughout the care system, up to 24 months post-injury, making sure they stay connected to services, treatments, and resources to help continued recovery of the Veteran with TBI [26]. Functions of the RSS include finding and facilitating access to TBI and psychological health care, supporting Veteran reintegration, and providing emotional support and referrals to in-person or online support groups [23]. These services and information are provided as a response to questions asked during phone calls at one month and every third month thereafter post-injury. Phone call questions cover a number of domains including physical symptoms, cognitive difficulties, psychological health, and psychosocial problems which are documented.

Other resources have been developed in the last decade to increase support to Veterans [23,27]. While this resource and the previously mentioned educational websites are important resources, they cannot fill all the need for education and support.

To meet these challenges this study investigates the perceived usefulness of available services and health information resources for Veterans and caregivers. We examine how these resources and services can be improved through self-reported experiences and preferences. These descriptions may help to advance the utility of health services for those providing care to Veterans with TBI and Veterans themselves. Additionally, these findings could assist in identifying gaps in service use, thus, guiding TBI health information and needed service research and development.

While there is growing research on reintegration for Veterans, to investigators’ knowledge, this is the first study combining qualitative interviews from both Veterans and caregivers of Veterans with TBI on their experiences and preferences of health information and service use. This study aims to provide detailed and comprehensive information on health service and information utility as well as preferred information and services in an ideal health model.

## Methods

### Research team

Interviewers were trained to conduct semi-structured interviews by a medical psychologist (AAW). Four interviewers conducted all interviews, 3 were Masters of Public Health graduate students, and one was an occupational therapist. All interviewers were female. Two interviewers were also contributing authors. No prior relationships were established with participants. No details about the interviewer was provided to the participants. All respondents were compensated with a $75 gift card for their participation in the interview.

Participants were informed researchers were interested in learning about the health information needs of individuals with TBI and how to improve them. Participants provided oral consent prior to beginning the interview. The oral consent process, interview guides and the research protocol were approved through the institutional review boards of the American Institutes for Research and George Mason University. For this research, an oral consent was approved by the institutional review boards because there was not an in-person meeting with the participants, but rather interviews were conducted over the phone. A written consent would have required that consent forms be emailed, mailed, or faxed back and forth. It was considered a risk to confidentiality to send signed consent forms in these manners. Therefore, we read the consent form to the participants over the phone and then if they wanted to, they provided consent to participate in the study orally.

### Study recruitment

Participants were recruited via various advertisement internet sites such as Craig’s List. Additionally, advertisements were posted on Veteran and caregivers of Veterans support group websites. All participants had to be over 18 years old. Participants had to self-report either having sustained a TBI while serving on active duty or caring for someone who sustained a TBI while serving on active duty. For this study, we defined TBI as an injury caused when an external force from an event such as a fall, assault, motor vehicle accident, or explosive blast injures the brain and causes loss of consciousness or a loss of memory. All participants reported that they had experienced this type of injury or were caring for someone who had experienced this type of injury.

### Study design

Interviews were conducted in a semi-structured format to obtain information regarding access to and utilization of health information, as well as the participants’ ideal health system/information model regarding receiving information related to the injury. This study was developed using the framework analysis approach [28]. This approach is within the broad family of analysis methods often termed as thematic analysis or qualitative content analysis. Interview guides were developed for Veterans and caregivers of Veterans with TBI. The guides were standardized for each group, but varied slightly between groups to tailor the interview to the target population.

The open-ended interview guides for both groups discussed participants’ individual experiences since the injury or since becoming a caregiver. Open-ended questions can be found in Table 1. Interviewers asked scripted questions on changes in health status, their primary information source(s) and subsequent perceptions of these sources, experience accessing services and the adequacy of services, and ideal health information and service system model.

**Table 1:** Semi-structured interview prompts.

Veteran Questions
1. Thinking back over the time since your injury, what has been most difficult for you?
2. What has been the most helpful for you since the injury in addressing the difficulties you mentioned
3. Overall, how do you feel that your general health has changed since becoming injured?
4. How do you currently receive information related to TBI?
5. How do you use the information you find/receive?
6. What are the biggest difficulties you face in getting the information you want about your condition?
7. How has your healthcare and social service providers been working together to provide your care?
8. I want you to imagine that we can start from scratch and develop a new approach [way] for getting information to individuals who have an injury like yours. In your dream world, what would this new model look like?
Caregiver Questions
1. Thinking back over the time since your [spouse’s, parent’s, child’s] injury, what has been most difficult for you?
2. Since the injury, what has been the most helpful for you in addressing the difficulties you mentioned?
3. Overall, how do you feel that your general health has changed since becoming injured?
4. How do you currently receive information related to TBI?
5. How do you use the information you find/receive?
6. What are the biggest difficulties you face in getting the information you want about your [spouse’s, parent’s, child’s] condition?
7. How have your healthcare and social service providers been working together to provide care?
8. I want you to imagine that we can start from scratch and develop a new approach [way] for getting information to caregivers of Veterans with TBI. In your dream world, what would this new model look like?

Caregivers or Veterans were in their homes for the phone interviews and are assumed to have been alone and/or otherwise responding without influence from other persons in the home. The caregivers interviewed were not related to the Veterans that were chosen. Detailed demographic information on all the participants can be found in Tables 2 and 3.

**Table 2:** Veteran demographic characteristics.

ID	Age	Gender	Service Branch	Years of Service	Years Since Injury
3301	27	Male	Army	8	5
3302	38	Male	Army	18	9
3303	25	Male	Army	3	1
3304	47	Male	Air Force	3	1
3305	40	Male	Navy	4	18
3306	69	Male	Air Force	4	47
3307	40	Male	Army	10	13
3308	55	Female	Navy	20	5
3309	41	Male	Air Force	22	10
3310	24	Female	Marine Corps	6	5
3311	41	Male	Air Force	5	23
3312	35	Male	Army	11	4

**Table 3:** Caregiver demographic characteristics.

ID	Age	Gender	Recipient Age	Recipient Gender	Years Since Injury
4001	46	Female	48	Male	18
4003	68	Female	38	Male	4
4005	32	Female	32	Male	1
4006	55	Female	54	Male	4
4007	45	Female	42	Male	1
4008	34	Male	36	Female	2
4009	40	Female	31	Male	2
4010	50	Male	29	Male	3
4011	35	Female	32	Male	4
4013	40	Male	29	Male	3

The interviews were conducted over the phone and averaged 55 minutes for interviews with Veterans, and 53 minutes for interviews with caregivers. Veteran interviews ranged from 34 to 82 minutes. Caregiver interviews ranged from 36 to 101 minutes. Interviews were audio-recorded and field notes were made during each interview.

### Qualitative analysis

Qualitative analysis was conducted on participants’ responses to the open-ended questions. We followed the framework analysis approach [28], a thematic analysis methodology to analyze the participants’ responses. To adequately analyze the qualitative data from the participants’ responses, a team of three researchers created a bank of responses from a random sample of four interviews for both caregiver and Veteran interviews. Separate banks were created for Veterans with TBI and caregivers of Veterans with TBI. The research team then reviewed and refined both answer banks. To verify the reliability of the answer bank, the research team coded an additional randomly selected interview that had not previously been used to define the answer bank. Coding discrepancies between the research team were then discussed, during which time working definitions of the codes were also created. The team of researchers (CK, SL, DP) then proceeded to code the 10 caregivers and 12 Veteran interviews using the refined answer bank.

Audio files were used to code for all interviews with the exception of one interview. The in-depth notes for this interview (taken by the interviewer during the interview) were used to manually code this interview, as the audio recording was unusable.

Some responses fell beyond the scope of the answer bank. In this situation, a new code was created and the research team was then notified of the addition of a new code. Overall, two new codes were added during the coding process for the caregiver interviews, and two new codes were added for Veteran interviews.

Qualitative data coding was completed using NVivo 10 for Mac software. To use advanced analytic features of NVivo, the project file was converted to the PC version on NVivo 11 for Windows. Through the PC version of NVivo 11, the frequency of each code was exported to a Microsoft Excel file. Using Microsoft Excel, the frequencies were used to calculate the number of participants of each group (caregivers and Veterans) that responded with any particular code.

Quantified codes for both groups were highlighted based on response frequencies. This guided the research team to identify themes that described the experiences of the participants which were then used to transcribe these portions of each interview. The results section highlights quotes from the interviews that are representative of the identified themes. Participants were not asked to provide feedback on findings.

## Results

A total of 22 participants were interviewed on the telephone: 12 Veterans with TBI, and 10 caregivers of Veterans with TBI (see Tables 2 and 3). The Veteran sample was predominantly male (83.3%), with a mean age of 40.2 ± 12.8 years. The majority of the Veterans were white (58.3%), with 25% of Veterans being African-American and 16.7% reported being of Hispanic descent. The majority were married or part of a committed unmarried couple (75%) with the others reporting being divorced or separated (16.7%). Veteran participants time since injury varied widely from 1 year to 47 years since injury with an average of 11.8 ± 13.0 years. The caregiver sample was predominantly female (70%) with a mean age of 44.4 ± 11.0 years. For the caregivers, 50% of the sample was white, 40% African-American, and 25% Hispanic. In terms of marital status, 37.5% were married or part of a committed unmarried couple, 37.5% were never married, and 25% were divorced/separated). For the Veteran’s that the caregivers were taking care of, the time since injury ranged from 1 to 18 years with an average of 4.2 ± 5.0 years.

The following sections discuss the results of the different topics as guided by the interview structure: experience since injury, health information sources and perceived usefulness, availability and adequacy of supportive services, and the ideal health information system. The main themes and sub-themes are presented in Table 4.

**Table 4:** Themes and sub-themes from qualitative analysis.

**Main Theme 1: Need for increases in personalized medical plan**		
**sub-themes:**		
Lack of coordination of health and social services (caregivers' & Veterans')	A need for more information on the specific injury (caregivers' & Veterans')	A need for information with less technical language to reduce time spent researching health information (caregivers' & Veterans')
**Main Theme 2: Need for an advocate or point person**		
**sub-themes:**		
Inadequate community support services (Veterans' & caregivers')	Lack of adequate transportation services (caregivers')	Lack of adequate hired in-home help (caregivers')
**Main Theme 3: Chronic nature of TBI**		
**sub-themes:**		
Reduced function is a major difficulty (Veterans')	Ideal service model includes 24/7 live support (Veterans' & caregivers')	Migraines is a major difficulty (Veterans')

### Experience since injury

Participants were asked about lifestyle changes they’ve experienced since the point-of-injury. The first section discussed what has been the most difficult and the most helpful since the injury. The most common response for Veterans who experienced a TBI was related to experiencing reduced function (n = 7). This was both physical (n = 3) and mental (n = 6). More specifically, experiencing frequent migraines, changes in memory, experiencing nerve damage, and sensitivity to light. These difficulties resulted in the need to quit work for one participant.

*Interview 3303 (Veteran)*: I'd say probably emotionally. Because after I got injured, I didn't know what I was going to do with my life, as far as my career. I had planned on being a career person for the military and with my future in the balance, emotionally I was distraught.

*Interview 3304 (Veteran)*: I had my injury a long time ago (1992) and I recovered pretty well from it. But, lately I’ve been having problems with my memory and executive functions and I have been suffering at my job.

*Interview 3308 (Veteran)*: Having to retire because I couldn’t work my job. That’s one and not being able to function or complete tasks the way I did before my injury.

*Interview 3312 (Veteran*): The thing I found most difficult is thinking about whether or not I could have done something differently to prevent or change the outcome.

Over half of caregivers cited witnessing patient’s struggles as the most difficult since injury. Increased responsibilities followed as the most difficult since injury for caregivers. Other responses included lack of support, lack of information or more stress.

*Interview 4005 (Caregiver)*: I’m the one that has to go to work now and, but, like, drive to those appointments that are usually far and you know traffic is horrible.

*Interview 4006 (Caregiver)*: Just that having to do a lot of things, you know he would have taken care of. Like calling somebody to do the yard and you know washing a car. Just like things around the house. Now, I have to try to find somebody and pay somebody to do those kinda things for me. He tries but it’s hard for me.

*Interview 4011 (Caregiver)*: It’s been difficult just seeing him not being himself and you know not being able to do the things that I know he wants to do. I’ve seen him before, and now. It’s just difficult to see him struggle like that. And also the Veteran Hospital, not giving him the help that he needs you know.

For more than half (n = 6) of caregivers the most supportive service since the injury has been their support network. The support network was most often the family of the caregiver and Veteran who experienced the TBI. Similarly, over half (n = 7) of Veterans interviewed also noted support system as the most helpful since the injury. Two Veterans specifically noted the American Legion as being the most helpful when providing further details on their support system. Several (n = 3) Veterans explained that staying active, being physically active or going to school, as the most helpful since the injury. Other responses from Veterans included their faith (n = 2) and that nothing has been helpful (n = 2).

*Interview 3302 (Veteran)*: The support system that I found through the American Legion has actually been extremely helpful. Talking to the older Veterans that have been through similar situations as me, and putting it in prospective. I think that's due to individuals at my local post that I go to.

*Interview 3304 (Veteran)*: My speech language pathologist has helped me a lot and has taught me coping mechanisms. Also, I have been getting help at the VA.

*Interview 3305 (Veteran)*: Being able to be physically active has been a huge help because I was very physically active before I lost my sight.

*Interview 4008 (Caregiver)*: Just kind of establishing a routine. You know the help of the staff at the rehabilitation center, optimize and figure out best practices, things we need to do at home, with certain challenges she has.

*Interview 4009 (Caregiver)*: The team of doctors they explain things to where I get it. If I don’t get it they will make sure that I do get it, to where I understand it completely and I think that’s a thumbs up for the team of doctors that he has.

#### Changes to health status

Nearly all responses from caregivers (n = 8) included experiencing either an increase in stress, anxiety, depression, and/or a need for counseling. Of these caregivers, close to half (n = 4) also described experiencing increased fatigue as a change to their health since becoming a caregiver.

*Interview 4008 (Caregiver)*: As far as energy wise, it just taxes a lot of you, so I’m not getting as much sleep. Not exercising as much, I don’t have as much energy, so I feel more tired.

*Interview 4011 (Caregiver)*: Yeah, I needed some counseling to help me through, because you know, that is my brother. So to see him go through this, I am in need of some counseling.

Veterans’ responses regarding changes in their health status were similar to their reported difficulties. The majority of the Veterans (n = 9) explained changes in their mental health, concentration, memory, increased irritability, and/or migraines as the most notable change in their health since the injury.

*Interview 3303 (Veteran)*: Since I was injured, I felt almost helpless because I had to have so much help for day-to-day activities. Um I would forget to eat. I would forget to take my meds. I would forget doctors’ appointments.

*Interview 3308 (Veteran)*: My mental health has changed. I physically can do whatever I want to do. I mentally can’t do everything that I want to do. So, my mental health has changed.

*Interview 3312 (Veteran*): I get migraines at least 3 times a week. I have two bulging disks in my back. I have minor nerve damage in my back.

### Health information sources and perceived usefulness

Many participants, both Veterans and caregivers, indicated their doctor was a main source of receiving health information (n = 7). Participants explained this was often used in combination with utilizing a variety of websites n = 8). The specific websites utilized by both groups were very similar. Most utilized websites and/or search engines such as Google, government websites (Department of Veterans Affairs), online videos on YouTube, and or WebMD. Other sources utilized for health information related to TBI were discussion boards and/or downloadable fact sheets. However, receiving health information from health care providers or a TBI specialist were strongly preferred over all other sources of health information, as this was viewed as the most trustworthy source of guidance by both groups.

*Interview 3303 (Veteran)*: Actually, a majority of the transition, like when I was going through the transition of being a soldier to being a civilian, I was assigned a AW2 advocate, which stands for Army Wounded Warrior advocate, and it's his job to keep me informed you know. Like, which VA I would go to, what health services they offer, and kind of guiding me where I need to go. I'm still in contact with him.

*Interview 4007 (Caregiver)*: It’s really hard to get accurate information if we are outside of his network of providers for his plan. So if we see family practice doctor or urgent care something like that I must be prepared to give full dissertation of his condition to them so that’s been difficult.

*Interview 4009 (Caregiver)*: With the seizures I did extensive reading up on how to calm them, how to look for signs that they’re gonna begin, cause you know he can’t tell me like, I’m about to, my head hurts, I don’t feel good, he just points to his head and squeeze his eyes and I know I gotta like, get him comfortable, lay him on his side so if it does come, I learned that from the internet. You have a more visual with the YouTube, you have a visual, more of a visual than reading step by step. So that’s what I had done in the beginning.

Caregivers often perceived web-based sources as either partially or not at all accurate (n = 6). In contrast, fewer Veterans perceived information to be partially or not at all accurate (n = 3). However, many caregivers explained information was either not at all or partially trustworthy (n = 4), pertinent (n = 5), or useful (n = 4). Similarly, Veterans perceived health information related to their injury or symptoms to be not at all or partially trustworthy (n = 4) or useful (n = 5). Veterans notably perceive information to be not at all or partially pertinent to their needs (n = 6).

*Interview 4007 (Caregiver)*: I receive my information directly from the physician and therapist. I just follow up with research on my own or I do Internet researches for terms and for things I am not familiar with.

*Interview 4010 (Caregiver)*: It doesn’t always relate to what specifically I’m going through. When it is exactly related to his condition it’s helpful, but if it’s just general support information helping the disabled person that’s not necessarily super helpful.

*Interview 4013 (Caregiver)*: There’s no specific name for or level for each of these guys and it fluctuates greatly. It’s hard to really peg it as one thing, because it could, it sounds like a million things. It’s hard to really get down to focus on what the real issue is, a lot of times.

Both caregivers (n = 5) and Veterans (n = 4) explained one of their greatest obstacles to obtaining needed health information was understandability of the information received or found via the Internet. This was often explained as overly technical language in information found via the internet or received from medical professionals. Many spent time researching definitions from the information they received. As such, time constraints were described as a common obstacle experienced by caregivers (n = 5) and Veterans (n = 4). This was further explained as limited time to research or limited time due to increased doctor’s appointments. For caregivers, time limitations were sometimes due to increased responsibilities. Some caregivers described needing specific information (n = 2) on symptoms or behaviors such as anger outbursts. Several Veterans reported being unsure where to obtain credible information (n = 4).

*Interview 3302 (Veteran)*: ..when they start using really big words I gotta break out a dictionary so I can get a definition of it. You know, sometimes the information online they, the way they have defined an injury like spindodiliosis, unless I look that up in a dictionary and try to find what the definition is I don’t understand what it was.

*Interview 3311 (Veteran)*: Because you’re not a doctor, you cannot understand half of what they’re saying. That would be the biggest issue. I don’t know what all this means. I have to continue looking, I have to figure out the wording. It is a big jumble.

*Interview 4003 (Caregiver)*: I have spent a large time on Internet hunting down information because, for example Veterans Administration in my area does not have a support group for families of TBI patients. It gave me a little limited information but I just had to do my own research on the Internet.

*Interview 4013 (Caregiver)*: I think you know, the information is not very up to date. There’s couple publications we get on a regular basis, but a lot of it is so general that it doesn’t really deal with us. Other than the internet, it’s pretty sparse out there.

### Availability and adequacy of supportive services

Over half of caregivers (n = 6) indicated that support for community participation (support groups) was not available and/or adequate. Hired in-home help had a noticeably higher response as not available and/or not adequate as well from caregivers (n = 4). Many caregivers also reported transportation was not available and/or adequate (n = 5). Veterans on the other hand were more likely to report these services to be available and/or adequate. Community support had the highest response of the services listed as not available and/or adequate from Veterans (n = 3). More often the interviewed Veterans indicated both availability and adequacy of the set of services except for community participation services (support groups) and hired in-home help.

*Interview 3302 (Veteran)*: I just started beating doors down until I got to the right service I know I needed. And asked a lot of questions. It was not handed to me. I had to track it down, demand it. Working with civilian contractors, doctors say oh you’re fine, these. No dude, you’re not hearing me. You’re giving me drugs, find me the help. Because the drugs aren’t always going to be there.

*Interview 3308 (Veteran)*: The only thing that I can say that was not adequate is support groups for a highly functioning disabled person that the medical professionals termed me. That was not something I termed myself. My thought process is if that the terminology that you used, then you should be able to find a support group for highly functioning disabled people because obviously I am not the first one.

*Interview 4001 (Caregiver)*: And but no one sat down and went over everything. At the time it was more detrimental to me because it was so scary. When you get information on paper and they have not written it and presented in a way that you know they make it sound like that this actually going to happen. And you know it doesn’t necessarily happen that way and you know they gave me no resources.

*Interview 4003 (Caregiver)*: Yeah just to be able to offer support group for families and people who have traumatic brain injury, make them available to people who need it or want to be part of a support group. I’ve just seen with my local hospital group that was started by one woman, some people have been going to it 15, 20 years now and it’s their only resource it’s the only place where they can get questions answered and get support.

When dealing with such a severe injury, there are often many medical professionals, counselors, case workers, or social workers employed to assist the Veterans and caregivers regaining as much functionality as possible. Half of caregivers explained that there was little to no discernible coordination of care from their social services workers or case workers and their medical team (n = 5). Similarly, some Veterans described a lack of coordination of care between their social service workers or case workers and medical teams (n = 4).

*Interview 3302 (Veteran)*: If they would communicate with each other. Doctor A will stick you on a bunch of this, Doctor B will say ‘Oh, well he’s an idiot. You need to be on this.” You don’t know who to listen to. You know, if they would consult with each other within that building they all work in, even though they are in different departments, if they would just pick up the telephone and call one another, and talk to one another, a lot of that stuff would be alleviated.

*Interview 4001 (Caregiver)*: There’s no interaction on the information that you receive. So there was a lot of just trial and error on my part. It’d have been nice you know if it was a perfect world and it’d be nice that you have you are assigned a traumatic brain injury social worker for the rest of your life basically.

*Interview 4013 (Caregiver)*: Just when we originally, when we took him home from the hospital there’s like a counselor that can get you started with a lot of local help… Well she, a lot of this information, is basically she starts you here and she says you need this you need that you need this, these are people to start with and they can kind of direct you to someone else that may be able you. She got us hooked up with a grant this program that will help pay for a modified car.

### Ideal health information system

When asked to describe their ideal health information model, both caregivers and Veterans had responses that highlighted a desire for an advocate or point person to guide them on finding services available to them. Others identified support groups as a necessary service in this ideal model.

*Interview 3301 (Veteran)*: The healthcare is excellent but unfortunately, it’s not easy because without good advocates there’s always a fight to get something. Not with the military, that’s with the VA. Got good care at both… I’ve had a little bit of both but I can’t complain.

*Interview 3304 (Veteran)*: Support groups. It is helpful to hear other people describe their experiences and what they did and it would be great to see that written up on-line. That way, if I was too shy to go to a support group, I could read it on-line.

*Interview 4001 (Caregiver)*: I would say as soon as the brain injury happens you have, in the hospital, you are assigned somebody as the TBI specialist mainly there to walk the family through the processes that are happening. And listening and maybe plugging in services that are needed immediately. And documenting when information needs to be addressed with the family in the care and not just one hour visit you know when it happens: ‘I’m available if you need me.’ It needs to be more interactive thing…then if children are involved is making sure that child and the person that has the TBI is still trying to keep the relationship and really making sure that emotional needs of the child are met as well. The first year should very hands-on.

*Interview 4003 (Caregiver)*: I think the very first thing that is needed is a contact person and by that I mean somebody I can call that has an official capacity medical or otherwise where I can connect and I know who to call who can help me access resources and information. So a contact person would be number one rather than me doing all the searching.

Respondents from both groups emphasized they want an increase in information incorporated into a personalized long-term medical plan, this was especially prominent among caregivers.

*Interview 3302 (Veteran)*: From the point of injury, it would be able to screen, identify based off of your injury what you may need. Whether it would be psychological, physical therapy, orthopedic therapy, occupational therapy, chiropractic, specialty doctors, pain management… But if it’s not available or made available, where you catch the wrong doctor, we turn off and we walk away. And I see it time and time again.

*Interview 3303 (Veteran)*: After the diagnosis, having a personalized care plan and everything, for that particular person, sent them with a digital copy and a hard copy… Kind of tailor the care plan, specifically for that person and their injury and their needs.

*Interview 3304 (Veteran)*: I think that my family and I should have been given more information and resources when I was discharged from the hospital.

*Interview 3305 (Veteran)*: I mean we had to figure out for ourselves all the resources we needed to go to when I got injured because a lack of information and knowledge.

*Interview 4001 (Caregiver)*: I’d say that as soon as you are injured like I said there is a registry that the hospital puts you in and starts to assign finding local support, social worker whatever to take on the case and start feeding you information as timeline goes on or as you ask for it.

*Interview 4003 (Caregiver)*: Maybe it’s just simply something connecting with other people whose family member with TBI has been through similar experiences and again that’s where I ran into issues because I was contacted by wounded warrior project but that’s more social activities that they provide for families and members of the family that have TBI and I’m not interested in looking for social activities. I need information. I need information to process to help me understand how I can help my son so this contact person to help me find the resources I might need whether for myself or my son just seems to be the key thing.

*Interview 4007 (Caregiver)*: A list of common terms and definition. A list of you know what to expect next, information about you know possible personality changes, what to expect, and of course a list of physicians, therapists, and other medical staff.

*Interview 4008 (Caregiver)*: Help with in-home, getting your home set up, as far as, I guess that would be sort of rehabilitation. Social services, depending on the person, help with healthcare and getting rides to and from therapy, things like that. Um you know, just covering all those bases. Then support, support groups, letting them go and talk to other people with the same injury and talking with a therapist or psychologist, or whatever they may need depending on what’s going on.

*Interview 4009 (Caregiver)*: When you’re becoming a caregiver it’s real important that the doctors and the medical team explain thoroughly what it is that is required of you and to be make sure that you understand not just tell you ‘here this is it, this is what you do’ and go on about your business. I think that is a big, important factor that everybody’s on the same page, everybody understands what the plan, what the healing plan is and stick to it and if they do that from the beginning it will make it much easier on everybody including the patient… if you have somebody who can explain it to you to the lowest common denominator and make sure that you understand and also give you hands on, hands on time to do things instead of reading it from a paper.

*Interview 4010 (Caregiver)*: I’d like somebody to come to the house and give video demos of step by step what to do, worst case scenarios, best case scenarios, hands on training for equipment that might be helpful, just basic day to day activities but more of a personalized nature training, I think would be ideal. Coming back on a regular basis to make sure all my questions are answered and that we stay on top of things.

When asked how they prefer to access needed health information in an ideal health system, caregivers and Veterans expressed wanting to see an expert in TBI to explain their condition and what to expect. Some Veterans and caregivers strongly prefer to receive information in person. Additionally, there was a notable emphasis of Veterans desiring physical copies of health information.

*Interview 3302 (Veteran)*: I want to be physically present to receive. I like stuff that I can feel in my hands, booklets, pamphlets, information to read and carry with me, so when I have five minutes I can open it up and read about it in my car at a stop light or at lunchtime and I can walk out to my car and read information. I’m not internet friendly.

*Interview 3309 (Veteran)*: I think that the best thing is actually talking to an expert instead of a doctor that can kinda tell you everything. You know I mean that they actually have some body that has expertise in traumatic brain injury to be able to assist people in. and be able to try to move on and try to live a life without having all the issues that people get with these types of injuries.

*Interview 3311 (Veteran)*: If it came from a doctor, it would be help, it would be more credible from a doctor. I do not have to look everywhere else. If a doctor said, try this or go to this place, it would be better. If it is on the internet, that is a bonus as well because it is right there at your fingertips. You have somebody to call. Possibly, there is a chat site in there for a chat group. Chat room representative for those who do not feel comfortable talking on phone. Easy to navigate, that’s another one.

*Interview 4007 (Caregiver)*: I mean I wouldn’t mind to talk to a social worker about it as opposed to a physician. Because a social worker just has a different level of you know they have a community based understanding as opposed to a physician.

*Interview 4008 (Caregiver)*: I prefer to get it obviously from the doctors and nurses cause you know, don’t have a lot of time. So doing research on the internet and trying to find information and stuff like that is pretty tough.

*Interview 4013 (Caregiver)*: If I could imagine something perfect obviously. I’d hope that it will be kinda administered by VA association which some sort of interactive real time connection between people looking for service. A lot of stuff is just so dated and you see so much crap behind it that you don’t know what to believe, so I don’t know. Maybe some mobile application. Something that is very interactive.

## Discussion

This study reveals important insights into the realities Veterans and caregivers of Veterans have experienced since the occurrence of a TBI. In these interviews caregivers describe their experiences and provide insights about transitioning into the role of a caregiver while Veteran interviews described reintegration challenges from an acute health care setting.

Our research provides unique insight to the needs of Veterans recovering from a TBI, a highly prevalent injury from OIF/OEF [2,29]. Veteran participants described what has been the most difficult for them personally coping with TBI. Some described somatic symptoms while others described cognitive symptoms. Some also described psychological changes such as dealing with challenges, such as not being able to return to work or having to leave the service, as the most difficult. To counter these difficulties, Veterans utilize various support networks such as family, friends, and peers. These responses align with current research on the difficulties Veterans experience with reintegration and the elevated strain placed on the individual when TBI occurs [30,31]. Elnisky et al. describes the domains of reintegration as interpersonal relationships, societal structures, and community systems [32]. A recent prospective longitudinal cohort study examining Veterans and service members with TBI and reintegration related themes found low levels of community participation were associated with cognitive ability and motor skills among those with moderate/severe TBI [33]. Our interviews with Veterans support these findings by describing challenges that include connecting with family members and lack of community support, as well as feeling sufficiently supported through work or school reintegration services.

Veteran participants described their overall health changing since the injury. These changes included somatic, cognitive and sometimes psychological changes following injury. The description of these health related outcomes among Veterans were consistent with previous research on long-term health outcomes of services members who experienced either mTBI and poly-trauma, or moderate-to-severe TBI [10,34]. However, a unique insight from our interviews is the emphasis on the chronic nature of TBI due to the wide variation of times since injury. Our findings of health-related changes, difficulties, and greatest support, among caregivers are similar to previous qualitative research of caregivers of patients with TBI [35].

Participants described needing more support over the first year, however, they expressly needed continued support due to changes in symptomology and experiencing new symptoms even after many years passed. Lack of services like community support and hired in-home help were reported by caregivers. Participants also consequently reported increased stress as a result of taking on more responsibilities since becoming a caregiver. Past research has examined outcomes of the increased stress of becoming a caregiver which has suggested a causal link among available caregiver resources, stressors and poor health outcomes for caregivers of Veterans with TBI [19].

Caregivers expressly wanted to discuss findings from doctors and internet searches among one another, through support groups, and find these networks to provide significant support. These findings are similar to a recent qualitative study which assessed life after leaving an acute health care setting for individuals recovering from TBI. This study found participants desiring increased transparency with doctors and an increase in follow-up from medical professionals [36].

Interestingly, Veterans were less likely than caregivers to report inadequacy of services, with the exception of community support. This could be due to their subsequent caregivers providing needed supports. Thus, a limitation of this study is not having interviewed Veterans matched caregivers. Veterans described a need to connect with others who have this continued experience, even in the case of a being classified as highly functioning. Therefore, both caregivers and Veterans could benefit from an increase in supports groups specific to the level of functioning.

Participants provided valuable insight on the vulnerability they each experienced during this time of transition from an acute setting. Responses highlight the need for Veterans and caregivers to have an advocate or point-person to assist with navigating transitioning out of an acute healthcare setting. Participants identified that this point person would ideally assist in the identification of healthcare providers and experts in TBI as well as assisting them in finding local support groups appropriate to their level of functioning. Participants, both Veterans and caregivers, described experiencing an absence of these important sources of support upon leaving an acute health care setting. This absence resulted in participants feeling stressed and disconnected from available resources or what to expect.

In the case of caregivers of Veterans, many were unaware of what to expect from their loved one and how to appropriately respond to their needs. Thus, nearly all caregivers expressed wanting to have someone to check on them and ask how they were coping and provide necessary support. Veterans consequently described spending extensive amounts of time researching treatments for themselves. Thus, a point person would connect Veterans and their respective caregivers to assist with reintegration after injury through providing more information, finding local services, and community support. This point person would alleviate the disconnection between an acute healthcare setting and reintegration to daily life. The previously mentioned Recovery Support Specialist (RSS) program offered by the DOD and VA only extended services up to 24 months’ post-injury [26]. Ideally, this point person would guide them for an extended, potentially unlimited period of time. This is critical due the chronic nature of TBI and symptoms that can change over time along with the natural aging process.

Participants revealed difficulties in accessing the health information they need. Veterans expressed experiencing difficulties in accessing information they perceived as pertinent to their needs and their specific condition for a variety of reasons. Further insight from Veterans revealed this was often due to lack of information specific to the injury, outdated information, or not knowing where to find credible information. Additionally, both caregivers and Veterans often felt language and a lack of understandability was a barrier to receiving and utilizing information on TBI. Previous research has shown these barriers often add to the stress of increased responsibilities caregivers face, in return, increasing negative health outcomes for the TBI patient [37].

A personalized care plan could potentially address these issues. This personalized care plan could provide Veterans with more information pertinent to their specific condition. To be optimally useful for both groups, this plan would need to be in plain language as both groups expressed a need for information that used less technical terminology. There is evidence that an integrated, comprehensive approach for reintegration and functionality can be successful. In a cohort study that followed 44 Veterans and military service members who had experienced TBI, the integrated and comprehensive approach had highly effective and sustainable results [38]. However, the needs of these individuals can change over time due to the chronicity of TBI. This can be further complicated by chronic conditions that can emerge as individuals age. Thus, a personalized plan should continue for the lifetime of the Veteran with TBI as their needs change over time. Investigators recognize it is unrealistic to provide Physical Therapy, Occupation Therapy, etc., on a weekly or monthly basis for an unlimited period of time. Thus, future research should investigate the feasibility of providing long-term personalized care for Veterans with TBI.

### Strengths and limitations

Semi-structured interviews with caregiver and Veteran participants provided an in-depth view of their experiences accessing, receiving care, continuity of care, and health information use and preferences. Combining both perspectives was critical to understanding trends since caregivers play a critical role in the lives of the Veteran who have experienced a TBI. Given the size of the population of Veterans who are reported to have experienced a TBI and this sample size, the generalizability of these findings is limited. However, as the chosen methodology was qualitative, generalizability was not the aim of this study.

A limitation was the variation in years since injury between the caregivers and Veterans interviewed. The time since injury among caregivers was within the past four years with the exception of one participant. However, the years since injury for Veterans was greater. In addition, information on the severity of TBI (mild, moderate, severe) was not collected. Therefore, we cannot address the impact of severity of TBI on our conclusions. This is an important variable to consider in future research.

## Conclusion

Interviews were conducted with Veterans with TBI and caregivers of Veterans with TBI providing insight into changes in health status, difficulties, and desired health information and services. Both Veterans with TBI and caregivers of Veterans with TBI experienced changes to health status and experienced difficulties associated with coping with a TBI. Reported service needs among both Veterans and caregivers were an increase in community support (support group) and hired in-home help. Both groups identified desiring more information and a long-term personalized medical plan. Ideal services include an assigned advocate to assist with identifying local resources and periodic frequent check-ins. These findings highlight the challenges, and possible remedies, to assist caregivers and Veterans with reintegration and after injury.
